# Fatal drug overdoses in healthcare workers: A thematic framework analysis of coroner reports

**DOI:** 10.1111/add.70139

**Published:** 2025-07-22

**Authors:** Thikra Algahtani, Siobhan Gee, Aminah Shah, Bryn D. Williams, Hayley C. Gorton, Sarah Welch, Caroline S. Copeland

**Affiliations:** ^1^ Centre for Pharmaceutical Medicine Research, Institute of Pharmaceutical Science King's College London London UK; ^2^ Pharmacy Department South London and Maudsley NHS Foundation Trust London UK; ^3^ Department of Anaesthetics Royal Free Hospital London UK; ^4^ Pharmacy School Aston University Birmingham UK; ^5^ Retired Consultant Addictions Psychiatrist; ^6^ National Programme on Substance Use Mortality London UK

**Keywords:** drug‐related death, drug toxicity, healthcare workers, mental health, overdose, suicide

## Abstract

**Background and aims:**

Healthcare workers face specific vulnerabilities for drug overdose due to their unique access to medications, clinical knowledge and work‐related stress. This study aimed to understand the characteristics of fatal overdoses in healthcare workers with a view to providing guidance for preventative strategies.

**Design, setting and cases:**

We retrospectively identified cases in England, Wales and Northern Ireland reported to the National Programme on Substance Use Mortality between 2000 and 2022 where decedents were working or studying within a healthcare setting at the time of their death or had previously worked in healthcare.

**Measurements:**

Quantitative analyses were conducted to report summary demographics of decedents, the circumstances of deaths and the drugs involved. A qualitative thematic framework analysis was performed to identify and explore factors that may contribute to fatal drug overdose in healthcare professionals.

**Findings:**

In the identified cases, doctors were the most represented profession (48% of cases, *n* = 28/58) with opioids the drug class most often implicated in causing death (43% of cases, *n* = 25/58). Whilst there was scant evidence of recreational drug use in the identified cases (*n* = 3), hospital‐only medications prominently featured [propofol in 29% (*n* = 17/58); midazolam in 10% (*n* = 6/58); neuromuscular blocking agents in 9% (*n* = 5/58)]. Qualitative analysis identified seven themes including accessing drugs from the workplace, use of skills and/or equipment for intravenous drug administration, obtainment of private prescriptions, diagnosed mental health conditions, recent events likely to have negatively impacted mental health, chronic pain and self‐medicating and history of substance use disorder and/or overdose.

**Conclusions:**

The characteristics of fatal drug overdoses among healthcare workers in England, Wales and Northern Ireland appear to differ from those observed in the overall population of people who use drugs in the UK. To prevent such deaths, it is important that healthcare workers can access bespoke care and support tailored to the specific challenges that they face.

## INTRODUCTION

People working within healthcare may be subject to specific factors with regards to drug overdose because of a unique combination of vocational contexts. They often have direct access to a wide range of licensed medications including potent opioids, sedatives, anaesthetics and other controlled substances [1]; access which represents opportunity for their non‐clinical use and diversion [2, 3]. Furthermore, their training provides them with knowledge of these medications, and they are familiar with their uses, which—although crucial for patient care—also provide them with awareness of the potential for their non‐clinical use. Healthcare professionals also often face high levels of stress and burnout because of the demanding nature of their work [4, 5]. Long working hours, the emotional strain of constant exposure to patient suffering—termed vicarious trauma—and the inability to provide care through lack of resources —termed moral injury—can contribute to the development or exacerbation of mental health issues [4, 6, 7]. When combining these factors, some people may turn to using the medicines readily available to them within the workplace as a coping mechanism. This ready availability may also increase the possibility that a person will use these medicines in the context of a suicide attempt. Therefore, there are two types of liability in relation to fatal overdose: accidental overdose in the context of substance use disorder (SUD) and suicide by fatal overdose. These may co‐occur in some individuals.

Despite the clear potential for drug overdose, few studies have examined SUD in healthcare professionals. Existing research studies are predominantly surveys requiring healthcare professionals to self‐report SUD (e.g. Oreskovich *et al*., Sørensen *et al*. and Geuijen *et al*.) [8, 9, 10], or the analysis of training records to identify healthcare professionals who were engaged with SUD treatment programs, who were subject to disciplinary measures as a result of a SUD, or who died while in training or practice (e.g. Warner *et al*., McLellan *et al*., The National Confidential Inquiry into Suicide and Safety in Mental Health and Skipper *et al*.) [3, 11, 12, 13]. Although the majority of these studies estimate a low prevalence of SUD in healthcare professionals (between 0.3% and 3%), this is likely an underestimation. This is because of barriers for self‐reporting such as denial, stigma and fear of negative repercussions on career, familial and social life [10, 12, 14, 15] and a low degree of problem recognition among healthcare professionals in identifying their own SUD who then go on to seek help [9], which together may heighten the likelihood of drug overdose. Under‐reporting of problematic alcohol use in healthcare professionals has been estimated at 40% to 60% [16], therefore, SUD rates may be similarly subject to underestimation.

There are gaps in the literature on fatal drug overdose deaths in healthcare professionals with contextual information about the decedents and their circumstances. An understanding of these factors could guide preventative strategies. We, therefore, studied coroners' reports, which include narrative reports about the circumstances of death. We sought to identify factors that contribute to fatal overdose in healthcare professionals, both intentional and accidental and guide recommendations for harm reduction.

## METHODS

This study was not pre‐registered so the results should be regarded as exploratory.

### Database: The National Programme on Substance Use Mortality

The National Programme on Substance Use Mortality (NPSUM) (previously the National Programme on Substance Abuse Deaths) was established in 1997 to collate coroner reports on deaths related to psychoactive drug use other than nicotine or caffeine. Although deaths related to psychoactive drug use in combination with alcohol are recorded, deaths because of alcohol alone are not collated by the NPSUM. Coroners in England, Wales and Northern Ireland voluntarily report a death to NPSUM if psychoactive drugs were detected and/or implicated in causing the death or if the decedent had a SUD.

Coroners collate information from a variety of sources when carrying out their investigations, including statements from witnesses, family and friends; reports from first responders such as police and paramedics; the results of *post‐mortem* and toxicology investigations; healthcare records pertaining to the deceased from general practitioners (GP), hospitals and drug and alcohol services. Coroners submit summary reports to the NPSUM, which are then transposed into a database with structured fields. Because these reports vary in terms of format and length, they are sometimes analysed using qualitative methods such as thematic framework analysis [17, 18].

### Selection of reports

We included reports where: (a) the date of death was between 1 January 2000 and 31 December 2022; and (b) the person had been employed as a healthcare professional or was in a non‐clinical hospital role either at the time of their death or previously (i.e. retired, on administrative leave or had left the profession) or was a student studying to become a healthcare professional at the time of their death and was detailed as having access to controlled drugs. We excluded deaths if the person was a veterinarian or worked in another role at a veterinary practice.

**FIGURE 1:**
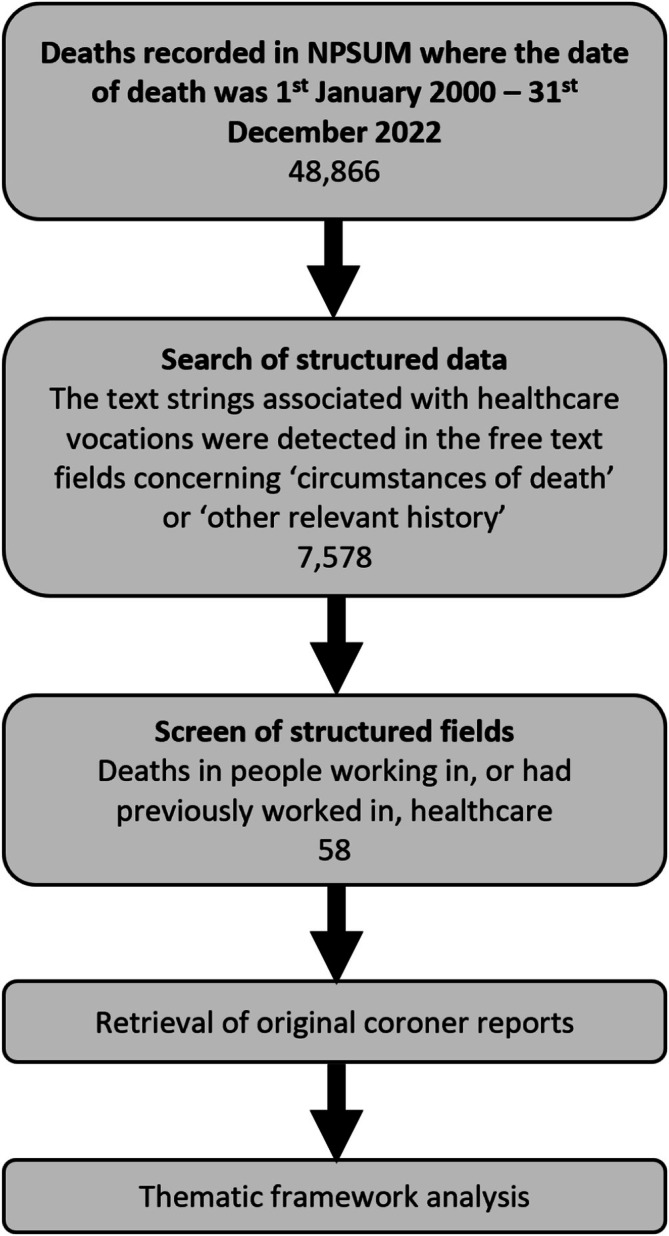
Flow chart of the process of selecting coroner reports for analysis. NPSUM, National Programme on Substance Use Mortality.

We identified relevant deaths following the process shown in Figure 1. We first filtered for cases where the decedent was listed as in active employment or a student when they died. We searched the ‘circumstances of death’ and ‘other relevant history’ database fields for text strings associated with healthcare worker vocations: ‘anaesth*’, ‘anasth*’, ‘cardiolog*’, ‘dentist’, ‘dermatolog*’, ‘doctor’, ‘endocrinolog*’, ‘GP’, ‘gynaecolog*’, ‘*medic*’, ‘nurse’, ‘obgyn’, ‘obstetric’, ‘oncolog*’, ‘orthopod*’, ‘paediatric*’, ‘pharma*’, ‘physioth*’, ‘ophthalmolog*’, ‘psychiatr*’, ‘radiogra*’, ‘rheumatolog*’, ‘surgeon’, ‘urolog*’. We screened the structured fields to exclude cases that did not meet the inclusion criteria. Two authors (T.A. and C.S.C.) double‐screened the sample with agreement of 99.8% and Cohen's κ of 0.98. We, then, retrieved the original reports and determined whether they met inclusion criteria.

### Analysis

We performed both quantitative and qualitative analyses. Quantitative analysis was conducted to report summary demographics of decedents, the circumstances of deaths and the drugs involved. We used qualitative thematic framework analysis [19] to identify and explore factors that may contribute to fatal drug overdose in healthcare professionals. Thematic framework analysis involved organising codes into categories developed by the research team. The codes were revised and updated as the analysis progressed [20]. We chose this method as the reports had inconsistent levels of detail—many with just a few words and some with several pages of narrative. The thematic framework analysis meant that each report could contribute. We also chose to use thematic framework analysis because we wanted to provide contextual information about decedents and their circumstances (drug overdose in healthcare professionals) rather than develop theory. In developing the thematic framework analysis, we used both an iterative deductive and inductive approach [i.e. a combination of building on *a priori* theory (deductive) and identifying themes from the data (inductive)]. Our deductive work was performed first and was informed by literature review of relevant articles on PubMed, Google Scholar and MEDLINE, existing concepts relating to ‘vocational factors’, and discussions among the research team before analysis. This provided a knowledge foundation before performing the iterative inductive work to develop the thematic framework analysis, which comprised of five stages: (a) three authors (T.A., A.S. and C.S.C.) independently open‐coded each case in triplicate. This involved adding tags and notes to the free‐text coroner reports; (b) we reviewed the codes together and created an initial thematic framework by identifying themes and grouping them into categories; (c) we applied the initial thematic framework to all cases with one author (T.A.) coding each case; and (d) we reviewed the results collaboratively and revised the thematic framework in several iterative rounds when potential new themes were identified.

We describe the thematic framework analysis, including representative quotes from coroners' reports. Although reports are publicly available (via Freedom of Information requests), we edited quotes to minimise the chance that an individual might be identified in our report. These edits included changing names, masking gender by use of they/them/their pronouns, replacing location names with fictitious names and altering the dates and times when events occurred.

### Ethics

The King's College London Biomedical and Health Sciences, Dentistry, Medicine and Natural and Mathematical Sciences Research Ethics Sub‐Committee re‐confirmed (August 2024) that analyses of NPSUM data do not require ethics review as all subjects are deceased.

## RESULTS

### Characteristics of cases

**FIGURE 2:**
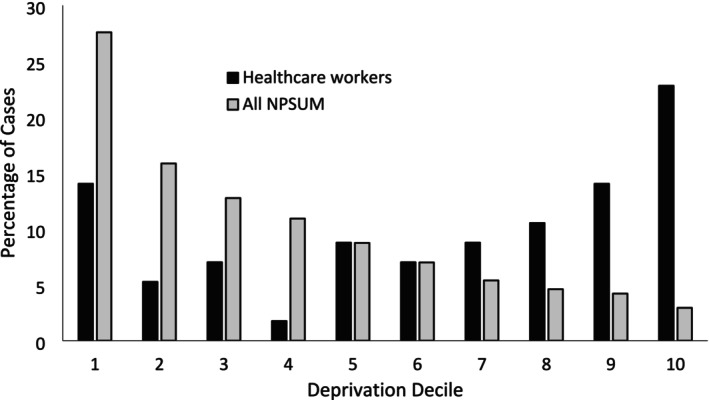
Deprivation deciles of usual addresses of healthcare workers. Data from all cases reported to the NPSUM have been included for comparison. Decile 1, most deprived; decile 10, least deprived; NPSUM, National Programme on Substance Use Mortality.

Fifty‐eight cases met our inclusion criteria: 47 people in active employment as a healthcare professional at the time of their death, three retired healthcare professionals, four healthcare professionals on long‐term administrative/sick leave, two non‐clinical hospital staff and two healthcare professional students who had access to controlled drugs. The median age of decedents was 38.0 (IQR = 31–47) and 67% were male (*n* = 39/58). Decedents were commonly listed as living with other people (55%; *n* = 32/58) rather than alone (40%; *n* = 23/58), with most having lived in more affluent areas of the country (Figure 2). The majority of people died in their own homes or in the room of their hospital accommodation (64%; *n* = 37/58). When people died in hospital (17%; *n* = 10/58) this was commonly cited as in a hospital toilet cubicle (70%; *n* = 7/10). In only one case, was the deceased found in the community and conveyed to hospital where they died despite treatment.

Doctors were the healthcare profession most represented (48%; *n* = 28/58); where practice specialty was known (70%; *n* = 19/27), anaesthetists accounted for 47% of cases (*n* = 9/19) and GPs for 26% of cases (*n* = 5/19). Death was deemed accidental in nature in 41% of cases (*n* = 24/58), suicide in 48% of cases (*n* = 28/58) and of undetermined intent in 10% of cases (*n* = 6/58). Accidental deaths predominantly occurred in males (88%, *n* = 21/24), with suicides also more prevalent in males (63% of cases concluded as suicide, *n* = 17/27). Opioids were the class of drug most often implicated in causing death (43% of cases; *n* = 25/58), followed by benzodiazepines (24% of cases; *n* = 14/58). Drugs seldom implicated in causing death in non‐healthcare professionals featured in this case cohort, namely the anaesthetic agent propofol in 29% of cases (*n* = 17/58), the short‐acting benzodiazepine midazolam in 10% of cases (*n* = 6/58) and neuromuscular blocking agents (rocuronium, atracurium, vecuronium bromide) in 9% of cases (*n* = 5/58). There were only three cases where illicit drugs were implicated in causing death—methamphetamine (ID 29954), cocaine (ID 27123) and cannabis (ID 69177)—and in all incidences were co‐implicated in combination with pharmaceutical medications. Characteristics of decedents are summarised in Table 1 and drugs implicated in causing death in Table 2.

**TABLE 1:** Demographic characteristics of cases.

Variable	Level	Total	
		*n*	% of cases
Total		58	100.0
Age	18–24	1	1.7
	25–34	23	39.7
	35–44	15	25.9
	45–54	15	25.9
	55–64	1	1.7
	65+	3	5.2
Sex	Female	19	32.8
	Male	39	67.2
Living arrangements	Lived alone	23	39.7
	Lived with family or friends	32	55.2
	Not known	3	5.2
Profession	Dentist	1	1.7
	Doctor	28	48.3
	Anaesthetist	9	15.5
	GP	5	8.6
	Medical student	1	1.7
	Psychiatrist	2	3.4
	Paediatrician	1	1.7
	Surgeon	2	3.4
	Retired	1	1.7
	Unspecified doctor	7	12.1
	Hospital operations manager	1	1.7
	Hospital porter	1	1.7
	Nurse (administrative/sick leave *n* = 4; retired *n* = 1)	16	27.6
	ODP	4	6.9
	Pharmacist (retired *n* = 1)	7	12.1
Manner of death	Accident	24	41.4
	Open (undetermined intent)	6	10.3
	Suicide	28	48.3
Place of death	Home	33	57.0
	Hospital accommodation	4	6.9
	Hospital	10	17.2
	Hotel/B&B	6	10.3
	Open space	3	5.2
	Not specified	2	3.4

Abbreviations: B&B, bed and breakfast; GP, general practitioner; ODP, operating department practitioner.

**TABLE 2:** Drugs implicated in causing death.

Implicated drugs	*n*	% of cases
**Alcohol**	**9**	**15.5**
**Anaesthetic agent (propofol)**	**17**	**29.3**
**Antidepressants** ^**a**^	**10**	**17.2**
Amitriptyline	2	3.4
Citalopram	2	3.4
Dothiepin	4	6.9
Mirtazapine	2	3.4
Sertraline	1	1.7
Trazodone	1	1.7
**Antiepileptic**	**2**	**3.4**
Lamotrigine	1	1.7
Sodium valproate	1	1.7
**Antihistamine**	**2**	**3.4**
Diphenhydramine	1	1.7
Promethazine	1	1.7
**Antihypertensives** ^a^	**2**	**3.4**
Diltiazem	1	1.7
Labetalol	1	1.7
Metoprolol	1	1.7
**Antipsychotics (olanzapine)**	**1**	**1.7**
**Barbiturates** ^**a**^	**5**	**8.6**
Amylobarbitone	2	3.4
Pentobarbitone	2	3.4
Thiopentone	2	3.4
**Benzodiazepines** ^**a**^	**14**	**24.1**
Alprazolam	1	1.7
Clonazepam	1	1.7
Diazepam	6	10.3
Flurazepam	1	1.7
Midazolam	6	10.3
Temazepam	2	3.4
**Cannabis**	**1**	**1.7**
**Cocaine**	**1**	**1.7**
**Gabapentinoids**	**2**	**3.4**
Gabapentin	1	1.7
Pregabalin	1	1.7
**Ketamine**	**2**	**3.4**
**Local anaesthetics (lignocaine)**	**3**	**5.2**
**Methamphetamine**	**1**	**1.7**
**Neuromuscular blocker**	**5**	**8.6**
Atracurium	3	5.2
Rocuronium	1	1.7
Vercuronium bromide	1	1.7
**Non‐opioid painkiller (paracetamol)**	**6**	**10.3**
**Opioids** ^**a**^	**25**	**43.1**
Alfentanil	2	3.4
Codeine	4	6.9
Dextropropoxyphene	4	6.9
Dihydrocodeine	1	1.7
Fentanyl	4	6.9
Methadone	1	1.7
Morphine	7	12.1
Pethidine	1	1.7
Tramadol	8	13.8
**Z‐drugs (zopiclone)**	**3**	**5.2**

*Note*: The bolded number is the number of cases with at least one compound within the drug class, but as in some cases, there were multiple, for example, for opioids, the total individual opioid detections sum to greater than the number of cases.

^a^ Multiple drugs were administered in some cases, so total number of implicated drugs will sum to greater than the total number of cases

We developed a thematic framework analysis consisting of seven themes organised into two categories: mental and physical health problems and vocational factors. Themes and categories are summarised in Table 3.

**TABLE 3:** Themes relating to drug overdose risk in healthcare professionals.

Category	Themes	No. of cases with history provided *n* = 42	Example case
Mental and physical health problems	Diagnosed mental health condition(s)	Yes: 29 None detailed: 13	The deceased was a community nurse who distributed prescribed medication to patients. They had previously been admitted to a psychiatric unit as they suffered with depression (ID 18910).
	Recent events likely to have negatively impacted mental health	Yes: 26 None detailed: 16	The deceased was a hospital nurse who had helped to treat patients after a terrorist attack and was suffering with PTSD (ID 22767)
	Suffering with chronic pain and self‐medicating	Yes: 6 None detailed: 36	The deceased was a hospital doctor who had been prescribed a weak opioid for a longstanding chronic pain condition. They were deemed to have self‐medicated with morphine stolen from work and accidentally experienced an overdose (ID 61459).
	History of drug misuse or overdose	Yes: 15 None detailed: 27	The deceased was a dentist who was prescribed oramorph, but would drink it recreationally. They were described to have drunk multiple ‘shots’ of oramorph on the day they died (ID 19233).
		No. of cases *n* = 58	
Vocational factors	Accessed drugs from the workplace	Yes: 37 No: 16 Not known: 5	The deceased was a pharmacist who had taken opioid, benzodiazepine and barbiturate medications from their workplace. This had not been prescribed to them (ID 5455).
	Used skills and/or equipment for IV drug administration	Yes: 32 No: 24 Not known: 2	The deceased was a nurse who was found with empty ampoules of pethidine, tramadol and diazepam around them along with a butterfly cannula (ID 15484).
	Obtained private prescriptions	Yes: 2 Not detailed: 56	The deceased was a hospital doctor who was found dead surrounded by multiple packets of prescription opioids that they had purchased in significant quantities from a number of on‐line pharmacies (ID 61878).

Abbreviation: PTSD, posttraumatic stress disorder.

The following section contains quotations from case studies that include sensitive material that some readers may find upsetting.

#### Mental and physical health problems

Four themes are Related to mental and physical health: diagnosed mental health condition(s); recent events likely to have negatively impacted mental health; suffering with chronic pain and self‐medicating; and history of drug misuse or overdose.

##### Diagnosed mental health condition(s)

The deceased were listed as having a history of at least one mental health condition in 69% of cases where a past medical history had been provided by the coroner (*n* = 29/42). The most prevalent condition cited was depression (*n* = 22). In a number of cases the deceased was described as having been in contact with mental health services in the days before their death, such as receiving mental health support from their place of work (IDs 29 954 and 61 744) or undergoing psychiatric assessment after recent worsening of their mental health (IDs 18 910, 22 596, 37 016 and 66 690).

##### Recent events likely to have negatively impacted mental health

In 64% of cases where a history was provided there were details of recent circumstances that could have negatively impacted mental health (*n* = 27/42). These included international moves (IDs 5455, 6345, 21 035, 59 400 and 61 878), splitting up with their partner (IDs 17 264, 17 448, 37 016, 48 035, 49 222, 55 965 and 59 400), exam stress (ID 6345 and 7190) and bereavement (IDs 19 233 and 70 017). In three cases, the healthcare worker died by suicide after being informed that they were subject to a professional misconduct (ID 43131 and 55 965) or criminal investigation (ID 27275). There was one case where a nurse had been placed on administrative leave because of a pending police investigation, became dependent on alcohol and then was deemed to have died following routine use of their antidepressant medication that proved toxic because of compromised hepatic function because of their excessive alcohol use (ID 17264). There were also circumstances that the deceased were exposed to because of their vocation that may have caused vicarious trauma or moral injury, such as ‘working 268 hours of overtime during the COVID‐19 pandemic’ (ID 65938) and treating victims of a terrorist attack (ID 22767).

##### Suffering with chronic pain and self‐medicating

Self‐medicating to relieve chronic pain was described in 14% of cases where a past medical history was provided (*n* = 6/42). In one of these cases, death was deemed intentional—the deceased suffered from a chronic pain syndrome that they ‘lived in constant fear of’ (ID 7190)—with the remainder concluded at inquest as accidental. Some decedents had obtained these drugs illegally from the workplace (IDs 7190, 49 547 and 61 459) or obtained them via private prescription (ID 69177). In others, the medications had been legitimately prescribed, but then used in risky ways, with one decedent known to regularly take more than their prescribed dose (ID 19233), and in another as administering their prescribed medication via ‘intravenous injection of drugs intended for oral use’ (ID 22767). Knowledge of the clinical pharmacology of these drugs may have led these decedents to believe that they could use them safely, but unknowingly administered a lethal dose.

##### History of drug misuse or overdose

Although a history of drug use was listed in 26% of cases where a past medical history was provided (*n* = 11/42), in some cases this history became apparent only after people had died. For example, in one case the decedent did not have a known living history of drug misuse, but there were ‘lesions on [their] wrist indicating IV drug use’ (ID 37478). In another case, the deceased's partner was aware of their drug misuse because they had tried to self‐manage opioid withdrawal at home on several occasions, but they were ‘too proud to get help’ from their GP (ID 61495). In other cases, the decedents were known to misuse drugs, with one decedent having been ‘given advice on safe medication and safe dosing’ by their GP (ID 69177), and another who was known to have ‘injected [their prescribed] codeine against medical advice’ (ID 22767).

Six decedents were known to have previously experienced an overdose on drugs: a nurse, who delivered patients their prescribed medication in the community, non‐fatally experienced an overdose with some of these prescribed medications 3 months before death (ID 18910); a student pharmacist, with access to medications as part of their training, non‐fatally experienced an overdose with medications taken from their dispensary 2 years before death (ID 19221); an anaesthetist, who non‐fatally experienced an overdose 9 years before death with anaesthetic agents taken from their workplace (ID 66690); a theatre assistant, who intentionally overdosed (but non‐fatally) in a hospital toilet cubicle with drugs taken from theatre 12 years before death (ID 70017); two cases—a nurse and a pharmacist—where no further details were provided about the previous non‐fatal overdoses (IDs 19 575 and 61 668). None of these decedents were listed as having a long‐standing history of drug misuse.

#### Vocational factors

Three themes related to the decedents' vocations: (i) accessed drugs from the workplace; (ii) used skills and/or equipment for IV drug administration; and (iii) obtained private prescriptions.

##### Accessed drugs from the workplace

Sixty‐four percent of the reports (*n* = 37/58) indicated that the deceased had stolen drugs from the workplace. These included decedents taking drugs home [e.g. ‘ampoules labelled vercuronium and propofol’ were located at one decedent's home (ID 7190)] or using stolen drugs while still at work. One decedent was found dead in a hospital toilet cubicle by their colleagues after their shift had ended, with an empty syringe that was later determined to contain midazolam and alfentanil (ID 37478).

For drugs that are subject to enhanced storage, supply and record keeping under the Misuse of Drugs Regulations (2001), covert measures would be needed to gain access to them without detection. This was illustrated by one case where ‘the anaesthetist had disposed of what they estimated to be the correct excess amounts of controlled drugs put out by the deceased (an operating department practitioner) that morning’ that indicated that the deceased ‘had topped up the medication they took with water or saline’ (ID 70355).

Thefts were not limited to healthcare professionals, as two reports detailed non‐clinical hospital staff gaining access to controlled drugs—a porter (ID 4510) and an administrative operations manager (ID 4361).

Reports indicate that some thefts were not one‐off events. In one case, the bottle of propofol that had been taken was of a ‘stronger concentration than the others’ subsequently found in the deceased's home (ID 65938). In two cases, there was evidence that pharmacists had repeatedly stolen medications from their dispensary (IDs 5455 and 19 575). In one of these cases, the pharmacist had been retired for a number of years and had used these retained medications to die by suicide (ID 19575).

##### Used skills and/or equipment for IV drug administration

Use of equipment from the workplace was reported in 55% of cases (*n* = 32/58). Specifically, the use of equipment to facilitate IV drug use such as ‘syringes’, ‘needles’, ‘cannulas’ and ‘tourniquets’ were mentioned multiple times. One decedent, who was an anaesthetist, was found unresponsive with a cannula in their hand that was connected to two bottles of anaesthetic agent (ID 59400). In another case, the deceased was a doctor who had taken non‐prescribed methadone into their workplace and used injecting equipment to fatally overdose (ID 61744).

Knowledge and skills because of vocation was also a recurring theme. Specific drugs may have been targeted because of the decedents' knowledge of their mechanism of action, physiological effects and toxicity profiles. These included potent opioids such as fentanyl (*n* = 4) and alfentanil (*n* = 2), the general anaesthetic propofol (*n* = 17) and insulin (ID 37016). There is also evidence of therapeutic polypharmacy to manage side effects of opioid drug use, such as the use of cyclizine or metoclopramide to combat the feelings of nausea (IDs 10 425, 22 717, 66 854 and 69 546). Employment of vocational skills to establish vascular access for IV drug administration will also likely have been a key factor in drug choice as many of the drugs implicated in causing death have low oral bioavailability (e.g. fentanyl, propofol, midazolam and insulin). Indeed, in one inquest conclusion the coroner commented that the decedent ‘would have had a good working knowledge of anaesthetic drugs used in surgical procedures and would have known that the drugs they injected themselves with would cause them to lose consciousness and stop breathing’ (ID 49222).

##### Obtained private prescriptions

In two of the 16 cases where the drugs implicated in causing death had not been sourced from the workplace, decedents had used private healthcare systems to obtain medications via private prescriptions. In one case, the deceased was described as a prolific user of on‐line pharmacies and was also taking ‘benzodiazepine which they were prescribed by a doctor in Bangladesh’ (ID 61878). In the other case, there was evidence that ‘the deceased was acquiring prescriptions overseas where they had been training to become a doctor’ (ID 69177). Knowledge of how to present a past medical history to qualify for certain drug prescriptions will likely have enabled decedents to influence prescriber decisions to obtain the drugs that they wanted.

## DISCUSSION

This study examined characteristics of fatal drug overdoses in healthcare professionals in England, Wales and Northern Ireland. Doctors were the most represented profession, and of these, anaesthetists prominently featured followed by GPs, surgeons and psychiatrists. Although this is reflective of vocational trends observed in a study of suicide involving any method among healthcare workers [21], the higher proportion of anaesthetists in this study may be explained by the focus on poisoning, as anaesthetists have ready access to potentially lethal drugs. A greater proportion of men than women died in this study, which contrasts with the findings of other studies evaluating suicides involving any method among healthcare workers [21, 22]. Again, this may be reflective of the study focus on poisoning (both intentional and unintentional), with drug use being more common in men than women [23], and that men are more likely to engage in risky behaviours [24]. Opioids were the class of drugs most often implicated in causing death—as is the case for overall drug‐related deaths in the United Kingdom (UK) [25]—but a variety of hospital‐only medicines featured, including anaesthetic agents and neuromuscular blockers. Again, this is likely reflective of the studied cohort as anaesthetists and other surgery‐related specialties have ready access to such drugs. Other demographic features for this cohort were distinctly different from those commonly observed in overall drug‐related deaths: all decedents were in active employment or studying for a healthcare degree at the time of their death, the majority were living with other people in affluent areas, there was scant evidence for recreational drug use and individuals were on average younger than the overall drug‐related death demographic with an average age of 39 (vs. mid 40s) [25]. Such a demographic will not usually be targeted by Drug and Alcohol Treatment Services for support. Finally, when applying the definition of suicide used by the Office for National Statistics (ONS) in England and Wales, that is intentional self‐harm in people age 10 and over and deaths of undetermined intent in people age 15 and over [26], then 59% of cases in this study are designated as suicide. This is nearly triple the NPSUM average of 21% [27].

This study further reiterates that healthcare workers face specific vulnerabilities with regards to fatal drug overdose because of their unique access to drugs, equipment and skills and their exposure to challenging and stressful conditions that may cause or exacerbate mental ill health. Some of these factors are inherent to the working environment, individuals or healthcare system and cannot easily be changed. However, other aspects may have scope for improvement to reduce fatal drug overdose in healthcare staff of the future.

### Prevention

There are already considerable measures taken to ensure that all drugs are accounted for in dispensing environments [28]. Healthcare professionals are fully aware of these necessary procedures, but at the same time, as demonstrated by cases in this study, therefore, know how these could be circumvented. It is not appropriate to suggest the introduction of further steps to monitor the dispensing of drugs and equipment that would hamper working practices. Rather, healthcare professionals need appropriate support to mitigate the occurrence of drug seeking behaviours.

Mental health support for healthcare workers should start at the very beginning of a career in healthcare. A worldwide review of self‐reported symptoms in medical students found one in four identified substance use [29]. Educational establishments delivering healthcare degrees must give greater emphasis to addiction and substance use, with a view to reducing drug use and promoting and encouraging early treatment and rehabilitation [30]. Provision of mental health support should continue for healthcare workers who have left the profession, as knowledge of drug action and administration are still present following cessation of active practice and may still contribute to vulnerability substance misuse. Although it could be assumed that access to drugs from the workplace would no longer be a vulnerability for such individuals, this study includes an incidence where medications stolen while in active employment were retained for subsequent use in a fatal overdose. Furthermore, mental health support should also be made more widely available across non‐clinical roles in healthcare environments, as this study demonstrates certain conditions (e.g. access to controlled drugs) may also affect these individuals.

Much has been published about resilience in the workplace, particularly since the coronavirus disease 2019 (COVID‐19) pandemic (e.g. Sotile *et al*. and Gerrard *et al*.) [31, 32]. A multitude of recommendations have been made to increase staff ability to cope, adapt and thrive in adversity, including intercollegiate support, supportive leadership and support for self‐care [33]. However, access for healthcare staff to mental health support can be challenging given the difficulties of accessing support during working hours, around shift patterns and fears over confidentiality or stigma [34]. For healthcare staff who do present to services, the possibility of deliberate or accidental poisoning should be considered given these individuals' unique access to knowledge and means. Accordingly, bespoke safety netting tailored toward healthcare workers is needed to prevent potential drug‐related harms, with specific consideration for those who may accidentally overdose and those who use drugs in the context of suicidal intent.

### Detection

Detection of missing substances is extremely challenging in busy healthcare environments such as hospitals with large and changing workforces. Interventions must be proportionate to the level of concern while still enabling effective delivery of patient care. Indeed, even with the stringent controls that are already in place, it is possible to circumvent detection methods by ‘replacing’ missing substances, as demonstrated in this study. Furthermore, the sheer volume of injecting equipment used daily in a healthcare workplace would make such items near impossible to track. A more effective detection strategy may be training programs to raise awareness in healthcare workers to recognise the signs and symptoms a colleague may display when they are facing difficulties [35], to understand how a person's role and working environment may be linked to specific challenges, and to know how to best provide them with appropriate support. Such an initiative could be integrated together with the delivery of drug use prevention strategies.

### Early intervention

Early intervention for staff experiencing difficulties with mental health, or with a substance use disorder, is key to improving outcomes. However, healthcare workers may be reluctant to seek help, as demonstrated in this study, often for fear of repercussions to their professional future [34]. Furthermore, although colleagues may be concerned about absenteeism, intoxication at work and poor work performance, they are often similarly reluctant to report problems [30]. An open culture for supporting staff and reducing stigma must be fostered by all healthcare communities. Clear supportive messaging from leadership teams would be needed for this to be realised. In this regard, following research that found that United States (US) physicians working in states where licensure applications overly probed mental health history were 20% less likely to seek help for a mental health condition, several US states changed the questions asked in their licensure applications [36].

A recent study found that one in three doctors experience suicidal thoughts during General Medical Council (GMC misconduct investigations [37]. Studies of registrants' experience of fitness to practice procedures of the UK Health and Care Professionals Council, which regulates most allied health professionals [38], and the General Dental Council, which regulates all dental professionals [39], have described a need for improved psychological support throughout the process. All professional healthcare bodies should schedule immediate in‐person support sessions for staff on commencement of misconduct investigations.

### Treatment and recovery

Although an extensive range of support services are available to healthcare workers [40, 41], they tend to postpone treatment until their drug use reaches a critical stage. This may be because of difficulties accessing treatment, not recognising that there is a problem until a late stage and fear of professional repercussions [34]. A mechanism to promote the help available to healthcare workers could be via a cubicle poster campaign in staff toilets, which—although directing readers to available support services—may also act as a point‐of‐decision prompt to discourage drug use in that space and to seek help. The promotion of visible exemplars of healthcare practitioners in recovery who have returned to work may also encourage those experiencing problems to seek help, as their demonstration of pathways to recovery may abate fears regarding their personal and professional futures and instil hope. The need for individually tailored back‐to‐work programmes is also needed and requires careful co‐ordination and monitoring [30].

### Limitations

The NPSUM receives voluntarily reports from coroners. Therefore, not all deaths in healthcare professionals from England, Wales and Northern Ireland will have been reported. Furthermore, we were only able to identify cases where the occupation of the deceased was specifically mentioned. The total number of fatal poisonings in healthcare workers has, therefore, likely been underestimated. Although the trends identified in this study are likely reflective of the true overall trends in fatal poisonings of healthcare, it is possible that further themes would emerge from cases that were not reported to the NPSUM, from those reported with insufficient detail regarding occupation or from those where the occupation was specified, but few further details regarding the circumstances of death or past history were provided. There is a field on the NPSUM form for coroners to report whether decedents were known to use drugs, but not a specified field as to whether there was known alcohol use disorder and were accordingly not able to report its incidence.

## CONCLUSIONS

The demographic profile of healthcare workers who died following drug use and the types of drugs they used are distinct from the typical demographics and patterns of drug use of people who use drugs in the United Kingdom. To prevent such deaths in healthcare workers from occurring, it is important that they can access bespoke care and support tailored to the specific challenges that they face. If substance use is identified, this should trigger the actioning of a support plan, whether within organisation (e.g. via occupational health) or externally by professional regulatory bodies when these become involved. When there is confidence in these processes, workers themselves and their colleagues may be more willing to act promptly. Although additional training to raise awareness of substance use and suicide in healthcare workers may add unwelcome burden on staff, healthcare professionals form a large proportion of the population and care for our own carers should be prioritised and promoted.

## AUTHOR CONTRIBUTIONS

**Thikra Algahtani:** Conceptualization (equal); data curation (equal); formal analysis (equal); investigation (equal); methodology (equal); project administration (equal); writing—original draft (equal); writing—review and editing (equal). **Siobhan Gee:** Investigation (equal); methodology (equal); writing—review and editing (equal). **Aminah Shah:** Data curation (equal); formal analysis (equal); writing—review and editing (equal). **Bryn D. Williams:** Investigation (equal); methodology (equal); writing—review and editing (equal). **Hayley C. Gorton:** Investigation (equal); methodology (equal); writing—review and editing (equal). **Sarah Welch:** Conceptualization (equal); data curation (equal); investigation (equal); methodology (equal); writing—review and editing (equal). **Caroline S. Copeland:** Conceptualization (equal); data curation (equal); formal analysis (equal); investigation (equal); methodology (equal); project administration (equal); resources (equal); software (equal); supervision (equal); validation (equal); visualization (equal); writing—original draft (equal); writing—review and editing (equal).

## DECLARATION OF INTERESTS

The authors have no interests to declare.

## Data Availability

Whilst the Data Protection Act does not apply to identifiable data that relate to a person once they have died, due to the sensitivity of the data used in this study the raw data will only be available upon reasonable request to the corresponding author.
